# The Deposition of a Lectin from *Oreochromis niloticus* on the Surface of Titanium Dioxide Nanotubes Improved the Cell Adhesion, Proliferation, and Osteogenic Activity of Osteoblast-like Cells

**DOI:** 10.3390/biom11121748

**Published:** 2021-11-24

**Authors:** Keicyanne Fernanda Lessa dos Anjos, Cynarha Daysy Cardoso da Silva, Mary Angela Aranda de Souza, Alessandra Batista de Mattos, Luana Cassandra Breitenbach Barroso Coelho, Giovanna Machado, Janaina Viana de Melo, Regina Celia Bressan Queiroz de Figueiredo

**Affiliations:** 1 Departamento de Microbiologia, Instituto Aggeu Magalhães (FIOCRUZ-PE), Campus da UFPE, Av. Prof. Moraes Rego s/n Cidade Universitária, Recife 50670-420, PE, Brazil; keicyanne1@yahoo.com.br (K.F.L.d.A.); cynarha@gmail.com (C.D.C.d.S.); maryaranda88@gmail.com (M.A.A.d.S.); 2 Centro de Tecnologias Estratégicas do Nordeste (CETENE), Av. Prof. Luiz Freire, 01. Cidade Universitária, Recife 50740-540, PE, Brazil; alessandrabmg@gmail.com (A.B.d.M.); giovanna@cetene.gov.br (G.M.); j.vianademelo@gmail.com (J.V.d.M.); 3 Centro de Ciências Biológicas, Departamento de Bioquímica, Campus da UFPE, Universidade Federal de Pernambuco (UFPE), Av. Prof. Moraes Rego s/n Cidade Universitária, Recife 50670-420, PE, Brazil; lcbbcoelho@gmail.com

**Keywords:** mannose-binding lectin, *Oreochromis niloticus* lectin, TiO_2_ nanotubes, biocompatibility, osseointegration

## Abstract

Titanium and its alloys are used as biomaterials for medical and dental applications, due to their mechanical and physical properties. Surface modifications of titanium with bioactive molecules can increase the osseointegration by improving the interface between the bone and implant. In this work, titanium dioxide nanotubes (TiO_2_NTs) were functionalized with a lectin from the plasma of the fish *Oreochromis niloticus* aiming to favor the adhesion and proliferation of osteoblast-like cells, improving its biocompatibility. The TiO_2_NTs were obtained by anodization of titanium and annealed at 400 °C for 3 h. The resulting TiO_2_NTs were characterized by high-resolution scanning electron microscopy. The successful incorporation of OniL on the surface of TiO_2_NTs, by spin coating, was demonstrated by cyclic voltammetry (CV), electrochemical impedance spectroscopy (EIE), and attenuated total reflection-Fourier transform infrared spectrum (ATR-FTIR). Our results showed that TiO_2_NTs were successfully synthesized in a regular and well-distributed way. The modification of TiO_2_NTs with OniL favored adhesion, proliferation, and the osteogenic activity of osteoblast-like cells, suggesting its use to improve the quality and biocompatibility of titanium-based biomaterials.

## 1. Introduction

Titanium (Ti) and its alloys have been extensively applied in the fabrication of implants and prosthesis to repair and/or replace hard tissues, due to their physical characteristics such as: high mechanical strength, corrosion resistance, and good biocompatibility [1,2]. Although, TiO_2_ presents several advantages, as low cost and improved biocompatibility over other biomaterials, therapeutic failure of TiO_2_-based implants and other medical devices may occur due to the ineffective bone formation and fixation, leading to bacterial infection and the implant loss. In this regard, the long-term success of titanium alloy implants is reliant on its stable fixation to the surrounding bone which, in turn, depends on the osseointegration—i.e., the formation of a direct interface between an implant and bone—without intervening soft tissue [3]. However, titanium *per se* lacks biological activity and cannot significantly promote cell adhesion and tissue healing. There is a consensus that the surface topography, morphology, chemical composition, and surface energy of titanium play a critical role on the cell adhesion, proliferation, production, and maintenance of extracellular matrix during the osseointegration process [4]. Therefore, physical and chemical modifications of the titanium surfaces have been developed to create more suitable interfacial microenvironment, promoting cell–material interactions and osseointegration [5,6]. Another way to improve the osseointegration can be achieved throughout the functionalization of implant material surfaces with biomolecules [7].

Lectins are non-immune proteins which bind reversibly and in a highly specific manner to simple or complex carbohydrates. These proteins have been widely investigated due to their prominent roles in several physiological and pathological processes, including immune response, inflammation, cell–cell communication, recognition, and differentiation [8]. It has been demonstrated that lectins from plants and animals have mitogenic [9,10], antibacterial [11,12], immunomodulatory [13,14], antithrombotic [15], and healing [16] activities. Some of these activities are remarkably interesting from the point of view of osseointegration and tissue repair.

The lectin OniL, from the plasma of Nile tilapia *Oreochromis niloticus*, is a mannose recognition lectin with mitogenic and immunomodulatory activities already proven in the literature [17]. These features make OniL a promising functionalizer of TiO_2_ surfaces. OniL is a C-type lectin that depends on Ca^2+^ for carbohydrate recognition. This class of lectins has demonstrated important roles in bone biology and pathogenesis. A C-type lectin domain family 11 member A precursor (Clec11a), also known as the stem cell growth factor (SCGF) or osteolectin 1, for example, has been identified as a growth factor able to promote the proliferation and differentiation of hematopoietic stem/progenitor cells [18]. The messenger RNA for this protein is abundantly expressed in proliferating chondrocytes, the primary ossification center, perichondrium, and periosteum [18]. Furthermore, it has been shown that secreted blood mannose-binding lectins (MBLs) of fishes, as well as other organisms, are important components of innate immunity, playing a crucial role in the body defense [17,18,19]. MBL is a constituent of the lectin pathway of complement system activation, one of the main components of innate immunity. This lectin plays an essential role in the defense against infectious microorganisms, maintenance of bone homeostasis, participating in different stages of bone healing [20].

Previous studies have demonstrated that a lectin from the seed of a leguminous plant, *Cratylia mollis*, was efficiently immobilized on the nanotubular surface of TiO_2_ nanotubes enhancing the adhesion of osteoblast-like cells [21]. In the present study, we explored—for the first time—the potential of an animal-derived lectin with mannose-binding specificity, as a coating agent of TiO_2_NT surfaces. For this the TiO_2_NTs are produced by anodic oxidation followed by thermal treatment [22]. The TiO_2_NTs were then negatively charged to improve the binding of OniL. The deposition of OniL on the surface of TiO_2_NTs was performed by spin coating. This technique is a solution-based process developed for low-cost deposition of thin films of molecules over a substrate surface [23]. The OniL-treated TiO_2_NTs were characterized and investigated for adhesion and osteogenic activity in osteoblast-like cells.

## 2. Materials and Methods

### 2.1. Materials and Reagents

Pure titanium was purchased from Realum Ltd. (São Paulo, SP, Brazil). For all the experiments, the Eagle’s minimum essential medium (EMEM), antibiotics, fetal bovine serum (FBS), were bought from Sigma-Aldrich Co. (St Louis, MO, USA). Rhodamine Phalloidin and 4′,6-Diamidine-2′-phenylindole dihydrochloride (DAPI) were bought from Thermo Fisher Scientific Co. (Walthan, MA, USA); isopropyl alcohol, ethylene glycol, ammonium fluoride, sodium hydroxide and ethanol were purchased from Merck Co. (Darmstadt, Germany). The human osteosarcoma cell lineage (HOS, ATCC^®^ CRL1543™) was purchased from ATCC Co. (Manassas, VA, USA). The kits for detection of alkaline phosphatase activity and calcium were purchased from LabTest Ltd. (São Paulo, Brazil).

### 2.2. Sample Preparation

Titanium samples (99.6% purity) with 0.5 mm thickness were prepared as square sheets sized 1cm^2^. The metal surface was polished utilizing a 400-grit emery paper down to 1200-grit emery paper, followed by wet polishing in a 15 μm alumina slurry. Next, the titanium samples were washed with distilled water, cleaned up with neutral detergent and sonicated for 10 min in isopropyl alcohol. Then, the samples were rinsed with deionized water and dried in a nitrogen stream. TiO_2_NTs were obtained by anodization of titanium samples by using a solution containing 89.3% ethylene glycol/0.7% ammonium fluoride in distilled water, as the electrolyte. The anodization was performed at a potential of 30V for 30 min at 2A. The time-dependent anodization current was recorded with a computer controlled Minipa ET-2076A multimeter (Minipa Co., Joinville, Brazil). After the electrochemical treatment, the samples were rinsed with deionized water for acid replacement and dried in a nitrogen flux. To crystallize the amorphous anodized TiO_2_NTs arrays into crystalline anatase phase, the samples were annealed in a furnace at 400 °C for 3 h under air atmosphere.

### 2.3. Coating of TiO_2_ with OniL

OniL was purified from the plasma of Nile tilapia *Oreochromis niloticus* and characterized as previously described [14]. For the lectin adsorption, the TiO_2_NTs were preincubated for 10 min in a 10% NaOH ethanolic solution to confer a negative charge to NTs surface (Neg-TiO_2_NTs). The OniL lectin, was then diluted at 100 and 200 μg/mL in PBS. Neg-TiO_2_NTs were coated with the lectin by spin coating, using a WS-650 Mz-23 NPPPB Spin Coater (Laurell Technology Co.,North Wales, PA, USA), operating at initial spin and final speed of 500 rpm and 2000 rpm, respectively, for 6 min.

### 2.4. Morphological Characterization of TiO_2_NTs

The morphological characterization of TiO_2_NTs was performed by high-resolution scanning electron microscopy, (Philips FEG/EDS QUANTA 200F) (FEI Co., Hillsboro, OR, USA) The binding of OniL on the surface of TiO_2_NTs was evaluated by cyclic voltammetry (CV), electrochemical 115 impedance spectroscopy (EIE), and attenuated total reflection-Fourier transform infrared spectrum (ATR-FTIR). The CV and EIE assays were performed using an Autolab potentiostat/galvanostat (Metrohm PGSTAT 128) (Metrhohm Co., Perdizes, Brazil). The Fourier transform infrared spectrum (FTIR) was recorded using a Bruker FT-IV spectrometer (Bruker, Billerica, MA, USA).

### 2.5. Cell Culture

Human osteosarcoma cell lineage (HOS, ATCC^®^ CRL1543™) was used as a model for adhesion, proliferation, and osteogenesis assays. For all the experiments, the cells (10^5^ cells/mL) were seeded on bare TiO_2_NTs or NegTiO_2_-NTs, coated or not with OniL (100 or 200 μg/mL), in 24-well culture plates containing 500μL of EMEM (0.5 × 10^5^ cells/mL) supplemented with 10% of FBS/1% penicillin/streptomycin, at 37 °C in 5% CO_2_ atmosphere for up 72 h.

### 2.6. Cell Adhesion, Proliferation, and Viability Assays

Cells cultured on the modified TiO_2_NTs, coated or not with OniL—for 24 to 72 h—were assayed for adhesion and proliferation. For this, the samples were stained with rhodamine-phalloidin fluorescent probe for F-actin and counterstained with 4′,6-Diamidine-2′-phenylindole dihydrochloride (DAPI) following the manufacturer’s instructions with minor modifications. Briefly, the cells were washed twice in prewarmed phosphate-buffer saline, pH 7.4, fixed in 3.7% formaldehyde in PBS for 10 min and permeabilized with 0.1% Triton X-100 in PBS for 5 min, at room temperature. After permeabilization, the cells were washed in PBS and staining with 5 µL of rhodamine-phalloidin methanolic stock solution diluted into 200 µL PBS/sample, for 20 min at room temperature. After the incubation time, the cells were washed twice in PBS, and counterstained with 300 nM of 4′,6-Diamidine-2′-phenylindole dihydrochloride (DAPI) diluted in PBS, for 3 min at room temperature. After incubation time, the samples were washed in PBS and visualized in a ZEISS Observer Z.1.apoTome microscope (Zeiss Co., Oberkochen, German). The number of adhered cells was estimated by counting the DAPI labeled nuclei in 10 randomly chosen field/sample (10X objective), using ImageJ 1.4.v software.

### 2.7. Osteogenic Potential

Cells cultured on the TiO_2_NTs were evaluated for its osteogenic potential. For this, after 24, 48, and 72 h of cultivation, the samples were washed three times in PBS and transferred for a new 24-well culture plates. The cells were lysed in a 0.5% Triton X-100 solution in PBS by three freezing/thawing cycles. The samples were collected, centrifuged at 10,000× *g* for 5 min and tested for ALP activity assay. ALP activity was quantified by the colorimetric alkaline phosphatase kit according to manufacturer’s instructions using para-nitrophenol phosphate as substrate. For calcium quantification the cells were cultured for 72 h, lysed as described above and submitted to calcium detection using calcium liquiform kit according to the manufacturer’s instructions. The absorbance of the samples was read at 590 nm for alkaline phosphatase and 570 nm for calcium detection, using a Multiskan GO spectrophotometer (Thermo Co., Walthan, MA, USA).

### 2.8. Statistical Analysis

The data are expressed as mean ± SD of two independent experiments in triplicate and analyzed by analysis of variance (ANOVA) test followed by Bonferroni post-test or Student *t*-test. *p* < 0.05 was considered statistically significant. The statistical analyses were performed using the GraphPad Prisma 5.0.

## 3. Results and Discussion

**Figure 1:**
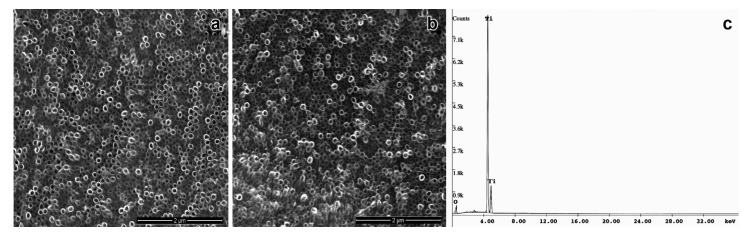
Ultrastructural assay of TiO_2_ modified nanotubes by high resolution scanning microscopy (**a**) bare TiO_2_NT, (**b**) OniL-TiO_2_NTs (200 µg/mL), and (**c**) EDS-spectrum of TiO_2_NTs.

Nowadays, modifications on the surface of titanium implants and its further functionalization with biomolecules have been investigated to improve the quality and biocompatibility of medical devices as prosthesis, and implants [6,7,21,22]. In this work, the anodization followed by thermal treatment of titanium surface efficiently produced a self-organized and homogeneous layer of TiO_2_NTs with a mean diameter 73.8 ± 8.2 nm, as confirmed by the EDS-SEM analysis (Figure 1a). Several studies reported that the viability, proliferation, migration, and differentiation of mesenchymal and hematopoietic stem cells [22,24,25,26,27], as well as the behavior of osteoblasts and osteoclasts [28,29] are strongly affected by the nanometric scale of TiO_2_NTs. The diameter of TiO_2_NTs obtained in our study was able to promote cell adhesion and proliferation, even in the absence of any functionalization of its surface. Our data corroborated previous studies that demonstrated an increase in the biocompatibility of TiO_2_NTs in 60–80 nm size range [25,30]. The annealing treatment after anodization changed the structure of TiO_2_NTs to the anatase, as observed by XRD analysis, according to the JCPDS file no. 21-1272. This crystallographic form of TiO_2_NTs has been reported as a highly organized structure that favors the nucleation of hydroxyapatite, the inorganic component of bone tissue, supporting the osseointegration process [7,31,32,33,34,35].

Although the electrochemical anodization is a useful approach to improve the biocompatibility and osteogenesis, as it provides an appropriate microenvironment for the fixation of bone cells, previous studies have demonstrated the great advantages of TiO_2_ surface modification with biocompatible and bioactive molecules [7,36,37,38,39]. This procedure has been shown to reduce the postoperative infection and improve the biocompatibility and osseointegration of the implant [7]. In this work, we used the lectin OniL to coat the surface of TiO_2_NTs. This lectin is a 17 kDa protein consisting of two subunits of 11 and 6.6 kDa. This protein presents a high affinity for methyl-α-D-mannopyranoside and D-mannose [14]. In mammals, mannose-binding lectins (MBLs) constitute a fundamental link between the innate immune system and other functions, such as coagulation, homeostasis after injury and defense against microorganisms [40]. As the success of the implant and prosthesis is intrinsically associated with the above-mentioned processes, we hypothesized whether the functionalization of TiO_2_NTs with OniL could improve the cell adhesion and proliferation, compared to the bare TiO_2_NTs. For this, we first incubated TiO_2_NTs with NaOH solution (pH 13) to charge them negatively improving the adsorption of OniL on its surface. According to Bavykin et al. [41] negatively charged nanotubes (Neg-TiO_2_NTs) promote electrostatic interactions with cations, presenting an excellent matrix for protein binding [42]. After being negatively charged, the samples were subjected to spin coating to immobilize OniL on the surface of Neg-TiO_2_NTs. The spin coating is a useful technique to fast and easily create a homogenous film with desired and well-controlled thickness [43]. As observed for the bare TiO_2_NTs the SEM analysis showed that the deposition of OniL on the surface of these nanotubes did not alter its morphology (Figure 1b). The elucidation of the chemical composition of these TiO_2_NTs by EDS showed the presence of Ti and O, demonstrating the successful anodization process, as well as the absence of sample contaminants (Figure 1c).

**Figure 2:**
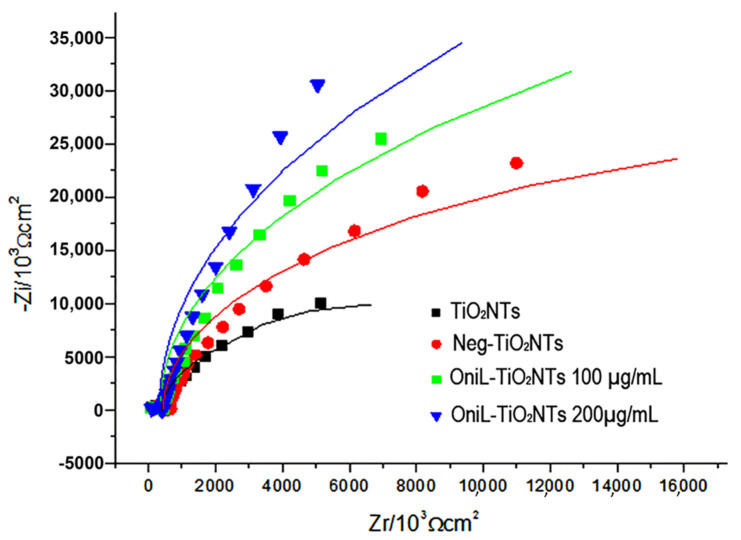
Nyquist plots of TiO_2_-modified surfaces. TiO_2_NTs (black line); Neg-TiO_2_NTs (red line), OniL-TiO_2_NTs 100 µg/mL (blue line) and OniL-TiO_2_NTs 200 µg/mL (green line).

The adsorption of OniL on the TiO_2_ surface was monitored by electrochemical impedance spectroscopy (EIS) using K4 [Fe(CN)_6_]/K_3_[Fe(CN)_6_] (1:1) as redox pair. The Figure 2 shows that each step of lectin immobilization generates a blockage in the transfer of electrons, increasing the resistance value (Rct). This result demonstrates that the lectin was efficiently immobilized on the surface of the samples. The bare TiO_2_NTs showed a Rct value of 1.7 kΩ. For negatively charged nanotubes, this resistance increased to 3.2 kΩ. The adsorption of OniL to TiO_2_NTs substantially increased the Rct value to 24.2 kΩ, whereas causing a simultaneous decrease in Cd to 6.89 µF. The impedance parameters, adjusted to the Randles equivalent circuit, are shown in Table 1.

The capacitive behavior observed in the Nyquist plots (Figure 2) was demonstrated by the presence of a double electrochemical layer at the electrode–solution interface, and the dielectric nature of TiO_2_ [44].

**Figure 3:**
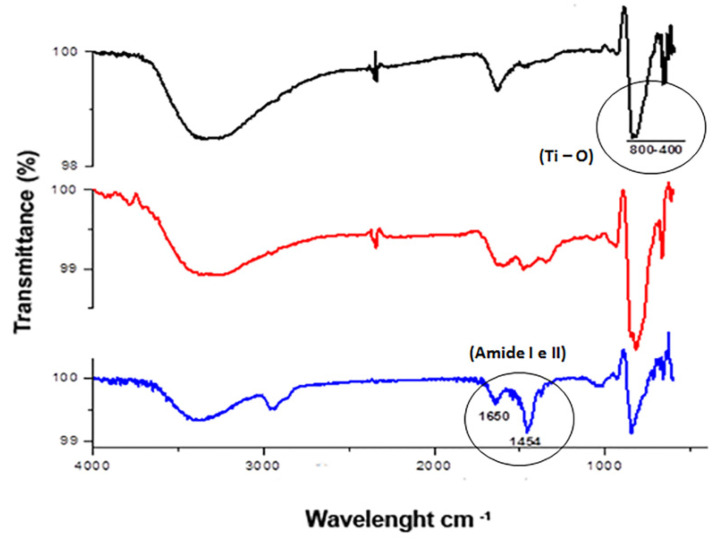
FTIR analysis of TiO_2_-modified surfaces. General aspect of the FTIR spectra of bare TiO_2_NTs (black line), Neg-TiO_2_NTs (red line), and NegOniL-TiO_2_NTs (blue line).

The ATR-FTIR analysis of TiO_2_ and Neg-TiO_2_NTs corroborated our electrochemical data. Our results revealed the presence of one absorption band peak characteristic of Ti-O vibration in the region of 400–800 cm^−1^. The presence of OniL lectin can be confirmed by the appearance of the two main stretches, corresponding to the lectin amide groups in 1643 cm^−1^ [45]/1025 cm^−1^ and 1456 cm^−1^/1010 cm^−1^ [46] (Figure 3). The secondary protein structures are usually identified by analyzing the vibration of amide I (1700–1600 cm^−1^), mainly due to the C=O elongation and amide II (1600–1500 cm^−1^), with minor contributions from C-N elongation and N-H [45,46].

In a previous work, the lectin Cramoll from seeds of *Cratylia mollis* bean was efficiently immobilized on the surface of anodized TiO_2_ nanotubes using layer-by-layer (LbL) technique [21]. This technique consists in the growth of alternated layers of poly (allylamine hydrochloride) (PAH) and poly(acrylic)acid (PAA). This self-assembling process occurs by the adsorption of oppositely charged polyelectrolytes on the surface of TiO_2_NTs [21]. Although layer-by-layer was proved to be useful to adsorb Cramoll lectin on the surface of TiO_2_NTs, this technique should be more expensive and time-consuming. In the present work, the lectin OniL was directly bound onto Neg-TiO_2_NTs without the need of any additional functionalization step, remaining strongly attached to the surface of negative charged TiO_2_NTs.

**Figure 4:**
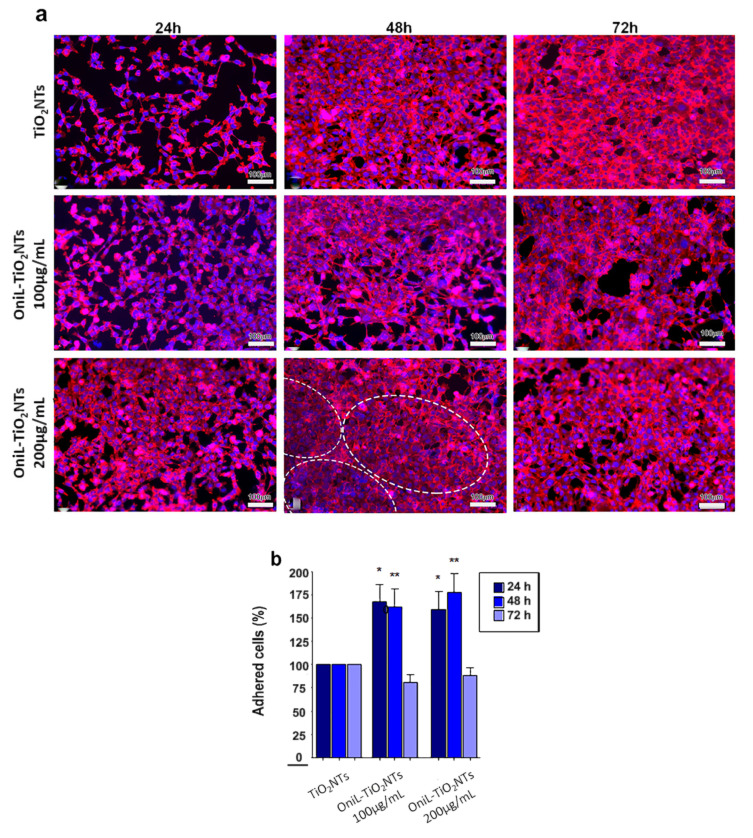
Biocompatibility analysis of OniL-TiO_2_NTs. (**a**) Representative fluorescence images of osteosarcoma cells adhered on the surface of TiO_2_NT after 24, 48, and 72 h of cultivation. The actin filaments and nuclei were showed in the red and blue channel, respectively. Note the concentric orientation of cells in the OniL-decorated nanotubes samples (dashed circle). (**b**) The quantification of cell nuclei in the DAPI-stained osteosarcoma cells. Significant differences compared to TiO_2_NTs for (*) 24 h and (**) 48 h of incubation, *p* < 0.05. The data were obtained from two independent experiments in triplicate.

The behavior of osteosarcoma cells in response to the OniL-decorated nanotubes was investigated (Figure 4). This osteoblast-derived lineage is widely used as a cell model to investigate the osseointegration on the surface of nanomaterials in vitro [47,48]. To evaluate the human osteosarcoma cells’ attachment on the TiO_2_-modified nanotubes, the cells were labeled with rhodamine-phalloidin, a fluorescent probe for actin, emitting fluorescence at the red channel. The quantification of the adhered cells was performed by counting the cell nuclei labeled with DAPI, which specifically binds to nucleic acids, emitting fluorescence at the blue channel. Our results showed that the deposition of OniL on the surface of TiO_2_NTs did not exhibited cytotoxicity and were able to significantly improve the cell percentage of adhered cells on the NTs. The osteosarcoma cells cultured on both the bare TiO_2_NTs and OniL-TiO_2_NTs (at 100 and 200 µg/mL) for 24 h showed a typical distribution of the actin filaments with the formation of focal adhesion points, an essential characteristic to maintain the shape, migration, and proliferation of the cells on the substrate [49]. At this time, there is a predominance of cells with spindle morphology and cell–cell interactions could be easily observed. However, the existence of empty spaces in the bare TiO_2_NT samples indicates a low rates of cell proliferation. After 48 and 72 h a confluent monolayer was observed in all TiO_2_NT preparations.

In the OniL-TiO_2_NTs samples, it was possible to observe that cells presented a more flattened extended phenotype and an increased spreading on the TiO_2_ surface. Interestingly, by 48 h of cultivation, the cells on the TiO_2_NTs decorated with 200 μg/mL of OniL begun to orient themselves in a more organized concentric manner in comparison to the those cultivated on the bare TiO_2_NTs (Figure 4a). Violin et al. [50], using the lectinhistochemistry methodology, evaluated the differential expression of surface glycoconjugates in a rabbit’s tibia implanted with microporous biphasic ceramic material. These authors showed that the lectin binding pattern during bone formation changed, corroborating the role of differential expression of glycoconjugates and its putative recognition by lectins during the osseointegration process. After 72 h of cultivation, the cell morphology remained preserved and strict cell–cell contacts could be observed in all modified TiO_2_NTs. The orientated organization of osteosarcoma cells observed on the OniL-treated surfaces, compared to bare TiO_2_NTs, may reflect what happens in vivo, showing the importance of carbohydrate recognition during osteogenesis on the surface of implants and prothesis.

The quantification of DAPI-labeled nuclei demonstrated a significant increase in the cell adhered to TiO_2_NTs in the samples treated with OniL for 24 and 48 h compared to the bare TiO_2_NTs (Figure 4b). The deposition of OniL on the surface of TiO_2_NTs was able not only to significantly increase the percentage of adhered cells by approximately 50%, but also stimulate their proliferation and differentiation on the TiO_2_NTs, in both concentrations tested, compared to the control group (TiO_2_NTs). After 72 h of cultivation, the percentage of adhered cells decreases in all samples at levels compared to the TiO_2_NTs (Figure 4b). This behavior is also observed in the human osteosarcoma cultures maintained in culture plates under standard conditions for up 48 h (data not shown). The decrease of percentage of adhered cells can be explained by the osteosarcoma cells commitment to osteogenesis rather than to the proliferation process.

**Figure 5:**
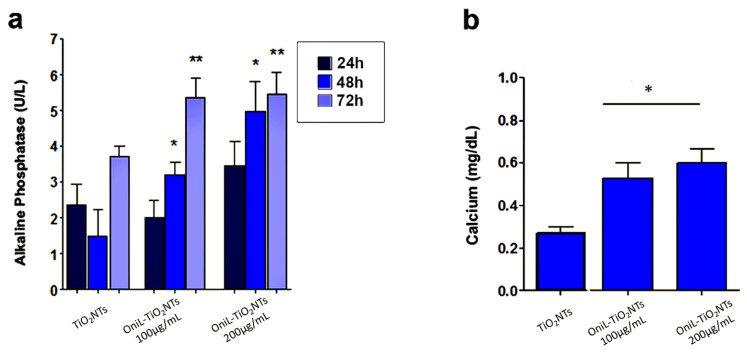
Osteogenic activity of osteosarcoma cells adhered to TiO_2_-modified surfaces (**a**) ALP activity (**b**) calcium quantification, after 72 h of cultivation. The data were obtained from two independent experiments in triplicate. Significant differences compared TiO_2_NTs, *p* < 0.05 for (*) 24 h and (**) 48 h of incubation.

To investigate whether the deposition of OniL on TiO_2_NTs was able to induce the osteogenesis, we examined the activity of alkaline phosphatase (ALP) (Figure 5a). Herein, we clearly demonstrated that the coating of NTs with OniL favored the rapid colonization of the substrate, allowing cell proliferation and osteogenic activity. Osteosarcoma cells cultured on OniL decorated nanotubes, showed a significant increase in the ALP activity as compared with TiO_2_NTs, mainly at 200 μg/mL of OniL. The highest increase was observed for OniL group after 48 h with values of ALP activity of 1.5 U/L (TiO_2_NTs), 3.2 U/L (OniL100 μg/mL), and 5.0 U/L (OniL200 μg/mL) (Figure 5a). A study by Ikeda et al. [51] showed that HOS cells cultivated on plastic culture plates presented ALP activity only after three weeks of cultivation. On the other hand, Min et al. [52] have demonstrated that the coating of TiO_2_NTs with laminin-derived functional peptides promotes HOS adhesion and ALP activity within the first 24 h of cultivation. Accordingly, we also showed that the modification of TiO_2_NTs with OniL was able to early trigger the osteogenesis processes in HOS cells. Taken together, these results showed that the adsorption of active biomolecules on the surfaces of biomaterials plays an important role in the induction of the osteogenesis process. Calcium, one of the main elements involved in the bone tissue remodeling, was also quantified after 72 h (Figure 5b). All the samples from the OniL groups presented a significant increase in the amount of calcium compared to the bare TiO_2_NTs samples. No statically significant differences could be observed between the lectin treatments (Figure 5b).

The osteoblasts cells are responsible for the synthesis, deposition, and mineralization of the bone extracellular matrix. In this process, the production of alkaline phosphatase is one of the parameters used to assess the effects of a biomaterial on the bone tissue activity [5]. The increased activity of this enzyme indicated the induction of the biomineralization process. In addition, during the growth and remodeling of adult bone, osteoblasts secrete calcium-rich vesicles to the calcifying osteoid [53,54]. As expected, the increase in ALP activity (Figure 5a) was followed by the simultaneous enhancement in the calcium deposition (Figure 5b) [54,55]. Both ALP activity and calcium deposition, observed in our study, are indicative of metabolically active and viable cells. Previous studies showed that mannose-binding lectins as *Lens culinaris* (lentil) lectin (LcL) and *Narcissus pseudonarcissus* (daffodil) lectin (NpL), a α-d-mannose-binding protein, did not elicit potent cytotoxicity against osteosarcoma cells [56]. Furthermore, a study by da Silva et al. [57] showed that OniL was able to favor the proliferation of Balb/c splenocytes without causing significant cytotoxicity to these cells. Furthermore, our fluorescence microscopy assay showed that the cells remained adhered to the surface of TiO_2_NTs, presenting a preserved morphology and nuclei integrity throughout the experiments. Although the improvement in the cell proliferation and adhesion on TiO_2_NTs seems to be a natural consequence of the ability of OniL to recognize carbohydrates on the surface of HOS, some issues—such as the speed of this process, the specificity of lectin, and mainly the fate of adhered cells after binding to the lectin—should be taken in account. Marty-Detraves et al. [58], for example, showed that the deposition of a lectin from the mushroom *Xerocomus chrysenteron* (XcL) inhibited the cell–substrate adhesion and proliferation of the adherent cell lines NIH-3T3 and HeLa cells, but not of the non-adherent SF9 cells. Interesting, XcL did not interfere in the cell cycle or induced apoptosis to these cells. On the other hand, the lectin from *Bauhinia forficata* (BfL) inhibited integrin-mediated adhesion of MCF7 human breast cancer cells inducing the cells to death [59].

Besides its role in the adhesion and differentiation of osteosarcoma cells, the deposition of OniL on the surface of TiO_2_NTs may have other beneficial consequences. Due to its immunomodulatory role, this protein can regulate the local inflammatory response, assisting in the bone healing and regeneration. Lectins can bind to the cell surface carbohydrates and trigger various cell events, such as stimulation of cell proliferation. In our study, we used an inexpensive and faster methodology to absorb OniL on TiO_2_NTs without the need for intermediate polymers.

## 4. Conclusions

In this study, we successfully functionalized TiO_2_NTs with the lectin OniL by using the spin coating methodology. The osteosarcoma cells cultivated on the surface of OniL-decorated TiO_2_NTs presented an improved adhesion and proliferation. The OniL also promoted an increase in both the deposition of calcium and ALP activity, which is indicative of enhanced osteogenic activity compared to bare TiO_2_NTs. The rapid colonization of HOS on the surface of OniL-treated TiO_2_NTs can prevent bacteria from forming biofilm on its surface, improving the chances of implant success. Although further studies are still needed to better understand the nature of OniL-TiO_2_NTs/osteoblast interactions, our results indicate that OniL could enhance the biocompatibility of TiO_2_NTs-based medical devices, assisting in the osseointegration between the bone and TiO_2_NTs surfaces.

## Figures and Tables

**Table 1:** Impedance parameters for TiO_2_NTs-modified surfaces using the equivalent cetesolution. The values were extracted from the parameters of the EIS equivalent circuit.

TiO_2_NTs Treatment	Cdl (µF)	Rct (kΩ)
TiO_2_NTs	38.89	1.71
Neg-TiO_2_NTs	35.33	3.30
OniL-TiO_2_NTs	6.89	24.20

Cdl, double layer capacitance; Rct, load transfer resistance.

## Data Availability

Not Applicable.
